# The Role of Psychosocial Support in Balance Improvements Following a Community-Based Tai Chi Program Among Latino Older Adults

**DOI:** 10.3390/bs16040573

**Published:** 2026-04-10

**Authors:** Zijian Qin, Shireen S. Rajaram, Carolina Padilla, Ka-Chun Siu

**Affiliations:** 1 College of Public Health, University of Nebraska Medical Center, Omaha, NE 68198, USA; qin.zijian@unmc.edu (Z.Q.); ssrajaram@unmc.edu (S.S.R.); 2 Intercultural Senior Center, Omaha, NE 68106, USA; carolinap@interculturalseniorcenter.org; 3 College of Allied Health Professions, University of Nebraska Medical Center, Omaha, NE 68198, USA

**Keywords:** elderly, Tai Chi, falls prevention, psychosocial support, ethnic minorities, physical activity

## Abstract

Falls are a leading cause of morbidity, loss of independence, and diminished quality of life among older adults, particularly in underserved ethnic minority populations. Physical activity interventions such as Tai Chi (TC) have been shown to improve balance and reduce the risk of falls. However, the influence of psychosocial factors in maximizing these benefits remains underexplored. This study examined the effectiveness of a community-based TC intervention and the role of psychosocial support in enhancing physical health outcomes among Latino older adults at risk of falling. Twenty-eight subjects were recruited to complete a 12-week TC program, and 23 participants with complete outcome data were included in the data analysis. Balance performance was assessed using the Timed Up and Go (TUG) Test at baseline, immediately after the 12-week intervention, and at a 12-week follow-up assessment (24 weeks from baseline). Psychosocial support was measured using the Norbeck Social Support Questionnaire. Linear mixed models were used to analyze changes in TUG scores and the moderating effect of psychosocial support. Results showed that participants with higher levels of psychosocial support showed significantly greater changes in balance than those with lower support scores (*p* < 0.05) immediately after the intervention program; these improvements were not maintained at follow-up. The findings should be interpreted cautiously, given the single-group design without a control group. Overall, the results highlight the importance of incorporating psychosocial components into health intervention programs for older adults, suggesting that supportive environments may be associated with improvements in both physical health and psychosocial well-being in aging minority populations.

## 1. Introduction

Falls in the geriatric population are a major public health issue in the United States and are the leading cause of both fatal and non-fatal injuries (Bergen et al., 2016; Qin & Baccaglini, 2016). According to the Centers for Disease Control and Prevention (CDC), approximately one in four adults aged 65 years and older experiences at least one fall each year, and about 37% of those who fall sustain an injury requiring medical treatment or restricting daily activity; collectively, falls among older adults account for more than 3 million emergency department visits and over 38,000 deaths annually in the United States (Centers for Disease Control and Prevention, 2024). Falls can result in hospitalization, disability, reduced quality of life, and mortality (Moreland et al., 2020). Furthermore, treatment of fall-related injuries, such as extensive physical therapy or surgery, can be highly costly (Bergen et al., 2016; Qin & Baccaglini, 2016; Reider et al., 2024).

According to 2024 Census population estimates, the U.S. population continues to grow older, in part due to the aging of the baby-boomer cohort. From 2023 to 2024, the U.S. Census Bureau reported a 3.1% increase in the population aged 65 and older (U.S. Census Bureau, 2025). With the elderly population growing steadily, the number of falls is expected to rise.

Latino individuals represent diverse racial backgrounds and originate from numerous Latin American countries. In the United States, Mexican Americans constitute the largest segment of the Latino population, which continues to grow, including among older adults. In 2019, there were 4.64 million Latino adults aged 65 years and older, representing approximately 9% of the nation’s older population; by 2060, this number is projected to increase to 19.9 million, accounting for 21% of all older adults (Administration for Community Living, 2021). The average life expectancy at birth is higher among Latino individuals compared to non-Hispanic White adults—81.3 years versus 78.4 years (Arias et al., 2025). This longevity advantage persists despite well-documented structural disadvantages, including lower socioeconomic status, reduced rates of health insurance coverage, and higher prevalence of comorbidities (Tarraf et al., 2020; González et al., 2019).

Tai Chi, a traditional Eastern mind–body exercise with more than a thousand years of history, is widely recognized as an evidence-based intervention for improving balance among older adults (Chewning et al., 2020). However, existing research on Tai Chi’s effectiveness has primarily focused on Chinese and non-Latino White older adults (Li et al., 2005; Wayne et al., 2014; Solloway et al., 2016), and only a limited number of studies have enrolled or reported outcomes for older racial/ethnic minority groups such as Latino older adults (Yang et al., 2021; Easwaran et al., 2020). More specifically, there is a notable paucity of data evaluating Tai Chi interventions among Latino older adults, a group experiencing substantial health disparities, including disproportionately high rates of falls (Easwaran et al., 2020; Sleet & Baldwin, 2014). Despite the demonstrated benefits of Tai Chi for fall prevention, Latino older adults remain underrepresented in many physical activity intervention studies and Hispanic adults report higher levels of physical inactivity compared with non-Hispanic White adults (Centers for Disease Control and Prevention, 2022). Barriers such as language differences, limited access to culturally appropriate programs, transportation challenges, and lower participation in structured exercise programs may contribute to this underrepresentation (Marquez et al., 2010, 2020). As the Latino population continues to grow rapidly in the United States, identifying effective and culturally responsive strategies to support healthy aging in this population is increasingly important (U.S. Census Bureau, 2025).

Psychosocial support constitutes a critical element in promoting health and well-being among individuals managing chronic illness or age-related physical and functional decline. Evidence shows that older adults who receive structured psychosocial support experience reduced depression, anxiety, and illness-related distress and demonstrate greater self-efficacy in managing their health (Niknejad et al., 2018; Akyirem et al., 2022). Psychosocial support also enhances motivation for maintaining treatment adherence and facilitates adaptive adjustment to changes in physical capacity, enabling older adults to reduce illness-related distress, maintain engagement in daily life, and sustain overall well-being (Uchino, 2006; DiMatteo et al., 2012). Within the broader psychosocial support framework, emotional support is characterized by empathy, understanding, reassurance, and relational warmth. Emotional support plays a fundamental role in buffering psychosocial stress and promoting resilience. Research has consistently linked adequate emotional support with improved mental health, reduced psychosocial distress, and enhanced quality of life among older adults and those living with chronic diseases (Devkota et al., 2023; Clausing et al., 2023; Asante & Tuffour, 2022). Also, insufficient emotional support is associated with higher levels of loneliness, mood disturbances, and frailty (N. Valtorta & Hanratty, 2012; Santini et al., 2020). Findings from these previous studies have highlighted the important role of psychosocial and emotional support within health intervention programs among older adults, particularly those disproportionately affected by chronic diseases. Psychosocial support may influence engagement with health promotion programs by shaping motivation, adherence, and sustained participation in physical activity interventions. In community-based programs such as Tai Chi, supportive social environments may reinforce participation and enhance the potential benefits of the intervention by fostering a sense of belonging, accountability, and confidence in physical functioning (Carron et al., 2012; Smith et al., 2017).

Given the demonstrated importance of psychosocial and emotional support in enhancing engagement in health-promoting behaviors among older adults, further research is needed to understand how these factors interact with culturally responsive interventions. This study aims to (1) evaluate the effectiveness of a Tai Chi intervention in improving health outcomes among Latino older adults, and (2) assess the extent to which psychosocial support contributes to participants’ engagement with and responsiveness to the intervention. We hypothesized that participation in the Tai Chi program would be associated with improvements in balance performance and participants reporting higher levels of psychosocial support would experience greater improvements in balance compared with those reporting lower levels of support. By examining these interrelated components, this study seeks to advance the evidence base regarding supportive, culturally tailored interventions for racial and ethnic minority older adults living with chronic health conditions.

## 2. Materials and Methods

### 2.1. Study Design and Participants

The study sample consisted of 28 Latino adults recruited from a local senior center in a Midwestern metropolitan area. Participants were recruited through community outreach efforts conducted at the senior center. Recruitment strategies included informational sessions, program announcements during community activities, and referrals from staff at the center. Interested individuals were screened for eligibility and invited to enroll in the Tai Chi program.

Eligibility criteria required that participants (1) were aged 50 years or older and able to follow verbal instructions in English; (2) had normal or corrected vision and hearing and no diagnosis of cognitive impairment, neurological or affective disorders, orthopedic or vascular conditions, or other comorbidities that could adversely affect balance or the ability to stand without support; and (3) completed at least eight of the 12 weeks of the Tai Chi intervention program and had both pre- and post-intervention measures of balance performance and psychosocial support.

Adults aged 50 years and older were eligible to participate in the program. Although many studies define older adults as individuals aged 60 or 65 years and older, community-based fall-prevention programs often include adults beginning at age 50 because mobility limitations, balance impairments, and fall risk factors may begin to emerge earlier in this population. Including participants aged 50 years and older allowed the program to reach individuals who may benefit from early fall-prevention strategies.

A total of 28 participants initially enrolled in and completed the Tai Chi program. For analytic purposes, participants were required to attend at least eight of the twelve intervention sessions and to have complete baseline and post-intervention balance assessments. Based on these criteria, 23 participants were included in the final analytic sample. Five participants were excluded due to incomplete balance assessment data or insufficient program participation.

The ability to follow English-language instructions was assessed during initial screening through direct interaction with research staff to ensure participants could safely engage in the movement-based intervention. The program was implemented in a bilingual community setting, with Spanish-speaking research staff available throughout recruitment, consent, and data collection to provide clarification and support as needed. All questionnaires were administered in English to maintain consistency. However, a validated Spanish version of the Norbeck Social Support Questionnaire (NSSQ) was available as a reference to facilitate accurate interpretation for participants who preferred Spanish-language support. This approach ensured participant safety during intervention delivery while promoting accessibility and comprehension of study materials. Data for this study were collected between November 2011 and August 2012.

### 2.2. Intervention Program

Following completion of baseline physical balance performance assessment, eligible participants enrolled in the Moving for Better Balance (MBB) Tai Chi program. MBB is a community-based, evidence-supported Tai Chi curriculum shown to improve balance and other health-related outcomes among older adults (Solloway et al., 2016). The program is designed for ease of dissemination and can be implemented effectively across diverse community settings.

**Figure 1:**
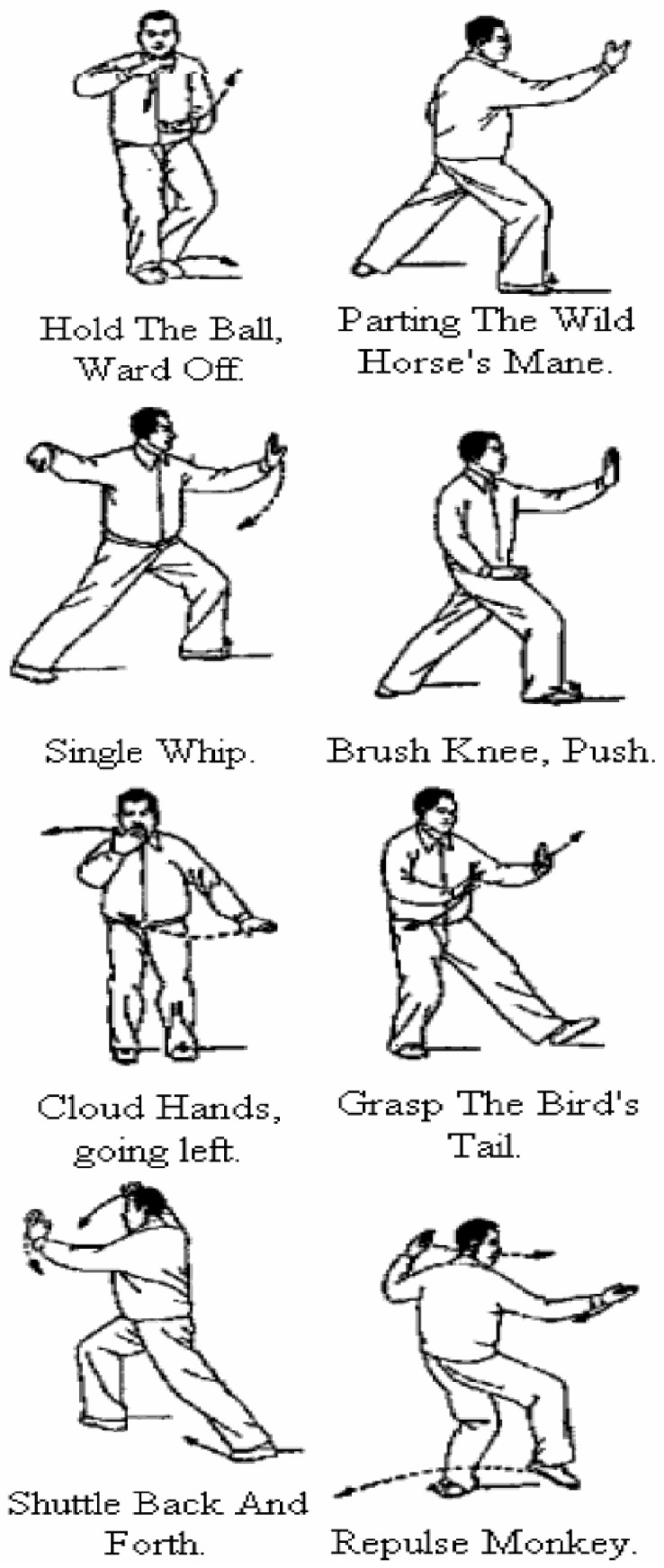
Moving for Better Balance (MBB) Tai Chi Instruction.

Participants were trained in eight standardized Tai Chi forms that emphasize core engagement and postural stability, progressing from foundational movements such as “Hold the Ball” to more advanced forms such as “Repulse Monkey” (Figure 1). Sessions were held twice weekly for 12 consecutive weeks and were led by a bilingual Latino Tai Chi instructor from the community who was trained and certified through the local chapter of the National Safety Council. The duration of each session varied depending on instructional content and participant needs. The MBB Tai Chi program mainly focused on the 8 forms instead of other standard programs with extended forms because our target learners were Latino older adults who were new to Tai Chi and needed substantial time to learn about Tai Chi. In addition, this 8-form Tai Chi program is evidence-based and has been shown to be beneficial in improving balance among older adults.

### 2.3. Balance Performance Assessments

Three balance assessments were conducted during the study. A baseline assessment was performed prior to enrollment to confirm participant eligibility for the MBB Tai Chi program. Two follow-up assessments were conducted after the intervention period. The POST assessment took place immediately after completion of the 12-week program to evaluate immediate effects on balance performance, while the FINAL assessment was conducted 12 weeks after the intervention, corresponding to 24 weeks from baseline, to assess the sustainability of any observed improvements.

### 2.4. Measures

#### 2.4.1. Outcome Variables

The primary outcome was change in balance performance across the three assessment periods—baseline, POST and FINAL. Balance was evaluated using the Timed Up and Go (TUG) Test, a validated functional mobility measure widely used in geriatric research (Mathias et al., 1986). The TUG requires participants to rise from a standard armchair, walk 3 m to a designated marker, turn, walk back, and return to a seated position. All TUG assessments were administered by a single trained research staff member to ensure consistency in test administration. Shorter completion times indicated superior balance and mobility. Change scores were calculated by comparing each participant’s post-intervention performance at POST and FINAL with their baseline values.

#### 2.4.2. Exposure Variables

The primary exposure variable was perceived psychosocial and emotional support, operationalized using the Norbeck Social Support Questionnaire (NSSQ) (Norbeck et al., 1981, 1983). The NSSQ is a validated self-report instrument designed to assess both the structural and functional characteristics of an individual’s social support system. Prior studies have demonstrated strong reliability and construct validity of the NSSQ across diverse populations, including older adults and clinical samples (Norbeck et al., 1983; Norbeck, 1995; Clingerman, 2004; Logsdon et al., 1996).

The NSSQ evaluates three interrelated domains: (1) social network; (2) functional support; and (3) relationship loss. Social network reflects the number and stability of individuals identified as meaningful sources of support. In this study, network support was quantified using a composite network score derived from the number of listed support persons, frequency of contact, and relationship duration. Because no established clinical cut points exist for this composite measure, participants were categorized into higher versus lower network support groups using the sample median (≥54 vs. <54). This approach is commonly used in NSSQ-based research to address differences in network size and to avoid averaging methods that may unintentionally reduce support scores for participants with larger networks (Norbeck, 1995; Gigliotti & Samuels, 2011).

Functional support is the primary psychosocial variable examined in this study, as it captures the extent to which individuals perceive receiving emotional, affirmational, and instrumental support from members of their support network. The emotional support component includes perceived empathy, encouragement, reassurance, and expressions of care. Functional support was operationalized using the NSSQ functional support scoring instruction (Functional support score = emotional + tangible support) and categorized using a median-aligned cutoff (≥100 vs. <100) to distinguish participants with relatively higher versus lower levels of perceived emotional and tangible support. To evaluate the robustness of our findings, a sensitivity analysis using a more stringent threshold (≥120 vs. <120) was also conducted to represent participants with particularly high levels of functional support (Norbeck et al., 1983; Norbeck, 1995; Logsdon et al., 1996).

Finally, relationship loss was assessed as the number of significant supportive relationships lost in the recent 12 months, reflecting potential disruptions to emotional and psychosocial support resources (Norbeck et al., 1981; Norbeck, 1995). For analytic purposes, relationship loss was dichotomized as no loss versus any loss.

We calculated Cronbach’s alpha coefficients to examine the internal consistency of the NSSQ support components, and they were high in the present sample. Cronbach’s alpha coefficients were 0.982 for social network support, 0.986 for functional support, and 0.812 for relationship loss score, indicating good-to-excellent internal reliability. Together, these exposure variables allowed for a multidimensional assessment of psychosocial and emotional support, encompassing both the availability of supportive relationships and the perceived quality of emotional support within the context of the Tai Chi intervention.

#### 2.4.3. Covariates

Covariates were included based on prior evidence linking them to balance performance in older adults (Bergen et al., 2016; Moreland et al., 2020; Mello et al., 2014). All covariates were self-reported and included age, gender, number of comorbid conditions, use of an assistive device for daily activities, and history of falls.

### 2.5. Statistical Analysis

We used linear mixed-effects models to evaluate changes in balance performance among participants across the baseline, POST, and FINAL assessment periods. First, univariate mixed models were fitted to estimate baseline TUG performance and subsequent TUG scores at POST and FINAL in relation to each exposure variable and covariate. A random intercept for each participant was included to account for within-person correlation across repeated measurements. The total time each participant spent practicing Tai Chi during the 12-week intervention was also included as a random effect to account for individual variation in program engagement.

We then constructed multivariate linear mixed-effects models by using balance performance changes as the dependent variable. Initial models included age and gender, followed by sequential addition of covariates that improved model fit. Covariate selection was guided by the Akaike Information Criterion (AIC) and by the statistical significance of crude associations identified in the univariate analyses. Model diagnostics were examined to ensure appropriate fit and adherence to regression assumptions.

In addition to reporting *p*-values and 95% confidence intervals, standardized effect sizes were calculated to facilitate interpretation of the magnitude of observed differences. For within-participant changes in TUG performance across time points, Cohen’s *dz* was computed based on paired differences. For comparisons between psychosocial support groups, Cohen’s *d* was calculated using pooled standard deviations of change scores. Because of the modest sample size, a descriptive post hoc power assessment was also conducted using the observed effect sizes for key comparisons. These analyses were intended to provide context regarding the statistical precision of the estimates rather than to serve as confirmatory hypothesis testing. Because the primary objective of this study was to evaluate balance outcomes among participants who engaged in the intervention and completed outcome assessments, analyses were conducted using data from participants with complete measurements rather than an intention-to-treat approach.

Although we hypothesized directional improvements in balance performance, all statistical tests were conducted using two-sided significance testing to maintain a conservative analytical approach. All statistical tests were two-sided, with an alpha level of 0.05 indicating statistical significance. Analyses were conducted using SAS version 9.4 (SAS Institute Inc., Cary, NC, USA).

### 2.6. Ethical Approvals

This study was conducted as part of a community-based Tai Chi program implemented under a broader research protocol approved by the Institutional Review Board (IRB) in 2011. The IRB approval year reflects the approval date for the parent research protocol under which the MBB Tai Chi intervention was conducted. The current study represents an analysis of data collected as part of this approved community health program.

## 3. Results

### 3.1. Baseline Characteristics

A total of 23 participants met the eligibility criteria and were included in the analytic sample. Participants ranged in age from 51 to 91 years, with a mean age of 65 years; 16 (69.6%) were female and 7 (30.4%) were male. Of the 23 participants included in the analytic sample, 21 attended all 24 Tai Chi sessions, representing full exposure to the intervention. The remaining two participants each missed a total of four weeks (eight sessions), with absences occurring across two different months. No injuries occurred during any training sessions.

Table 1 presents baseline TUG performance across different demographic and psychosocial characteristics. Participants aged younger than 60 years had a mean baseline TUG time of 13.18 s, compared with 13.00 s among those aged 60 years and older. Female participants demonstrated a mean baseline TUG time of 12.82 s, while male participants averaged 13.58 s. More than half of participants were classified as having higher social network support and functional support (52.2%) based on median-aligned cutoffs derived from the NSSQ. Baseline TUG performance did not differ meaningfully by social network score, functional support score, or relationship loss status. Approximately 30% of participants reported at least one fall in the previous 12 months, and those with a fall history demonstrated slower baseline TUG performance than those without a fall history.

### 3.2. Tai Chi Program and Overall Balance Performance

Across the full sample, participation in the 12-week Moving for Better Balance Tai Chi intervention was associated with improvements in balance performance, as reflected by positive changes in TUG scores immediately after the intervention and at the 12-week follow-up. Improvements were observed across most demographic and clinical subgroups, although the magnitude of improvement varied (Table 2).

Age group and gender were not significantly associated with either immediate or sustained changes in TUG performance in crude or adjusted mixed-effects models. Participants aged 60 years and older demonstrated modest improvements at the 12-week follow-up, but these changes were not statistically different from those observed among younger participants. Similarly, both female and male participants showed improvements in TUG performance following the intervention, with no statistically significant differences between genders.

Clinical characteristics, such as fall history and assistive device use, were not significantly associated with immediate balance improvement. Participants with a history of falls demonstrated larger improvements at the 12-week follow-up compared with those without a fall history; however, these differences did not reach statistical significance. Comorbidity burden showed a suggestive association (crude *p*-value = 0.051) with sustained improvement (at the 12-week follow-up), with participants reporting no comorbidities exhibiting the largest improvements, though overall differences across comorbidity categories were just borderline (adjusted *p*-value = 0.066) in adjusted analyses.

### 3.3. Psychosocial Support and Balance Performance

Findings related to the three NSSQ domains—social network support, functional support, and relationship loss—revealed positive patterns in relation to balance improvement among Latino elderly following the Tai Chi intervention.

Higher social network support was significantly associated with greater immediate improvement in balance performance. Participants with higher network scores demonstrated a mean improvement of 2.34 s in TUG performance immediately after the intervention, whereas those with lower network support showed an average negative change (minus 0.18 s). This association remained statistically significant after adjustment for age and gender. At the 12-week follow-up, participants with higher network support continued to demonstrate greater improvement. However, this statistical difference was no longer significant (Table 2).

Similarly, higher functional support was significantly associated with immediate balance improvement. Using the primary cutoff, participants with higher functional support (functional support score ≥ 100) demonstrated significantly greater immediate improvements compared with those with lower support in both crude and adjusted models. Because both the social network score and the primary functional support score were dichotomized using sample median cutoffs, the resulting high and low support groupings were identical in this dataset. Consequently, the statistical results for these two variables are the same in Table 2. When a more stringent cutoff (functional support score ≥ 120) was applied in our sensitivity analysis, the magnitude of both immediate and sustained improvements increased, and the association with immediate improvement remained statistically significant. Sustained improvement at 12 weeks was larger among participants with high functional support in sensitivity analyses, although this association did not reach statistical significance after adjustment.

Participants reporting no loss of supportive relationships demonstrated greater improvements in balance performance compared with those reporting one or more losses. Immediately following the intervention, participants with no reported relationship loss showed a mean improvement of 1.93 s in TUG performance (95% CI: 0.27, 3.59), whereas participants reporting relationship loss exhibited smaller changes (mean = 0.41 s; 95% CI: −1.03, 1.85). This pattern was evident in crude analyses and persisted after adjustment for age and gender, although the difference did not reach statistical significance (adjusted *p* = 0.083). At the 12-week follow-up, participants with no reported relationship loss continued to demonstrate greater sustained improvement in balance performance (mean = 1.81 s; 95% CI: 1.18, 2.45) compared with those reporting relationship loss (mean = 1.12 s; 95% CI: 0.45, 1.80). Differences between groups were not statistically significant in either crude or adjusted models (adjusted *p* = 0.344), but the direction of association consistently favored participants without recent loss of supportive relationships.

Standardized effect sizes were calculated to complement statistical significance testing. The overall improvement in TUG performance from baseline to the POST assessment corresponded to a moderate within-participant effect size (*dz* ≈ 0.40), while the improvement from baseline to the FINAL assessment corresponded to a moderate-to-large effect size (*dz* ≈ 0.60). For subgroup comparisons, participants with higher functional support demonstrated substantially larger improvements in TUG scores immediately following the intervention compared with those with lower functional support, corresponding to a large between-group effect size (Cohen’s *d* ≈ 0.98). Sensitivity analyses using the more stringent functional support cutoff also showed large effect sizes (*d* ≈ 0.87–0.88), although these comparisons did not consistently reach statistical significance, reflecting the limited statistical power associated with the small sample size.

## 4. Discussion

This study examined the effects of a community-based Tai Chi intervention on balance performance among Latino older adults, with particular attention to the role of psychosocial support. Consistent with existing evidence (Li et al., 2005, 2018; Low et al., 2009; Solloway et al., 2016), participation in the MBB Tai Chi program was associated with improvements in balance performance immediately following the intervention and at 12-week follow-up.

The observed overall improvements in balance performance align with a substantial body of literature supporting Tai Chi as an effective intervention for improving postural control and reducing fall risk among older adults (Li et al., 2005, 2018; Solloway et al., 2016). Previous randomized trials and community-based studies have demonstrated that Tai Chi enhances lower-extremity strength, coordination, and proprioception, leading to measurable improvements in functional mobility (Li et al., 2005; Wayne et al., 2014). Our findings corroborate these benefits within a Latino older adult population, a group that has been underrepresented in Tai Chi research (Siu et al., 2017; Easwaran et al., 2020).

Demographic characteristics such as age and gender were not significantly associated with balance improvement, a finding consistent with prior studies showing that Tai Chi confers benefits across a broad range of older adults regardless of age group or gender (Li et al., 2018; Wayne et al., 2014). Similarly, fall history and assistive device use were not significantly associated with immediate improvements, although participants with fewer comorbidities demonstrated greater sustained benefit. This pattern is consistent with the literature suggesting that multimorbidity may limit the durability of functional gains following exercise interventions due to reduced physiological reserve or competing health demands (Marengoni et al., 2011; Puthoff, 2008).

The most novel findings of this study relate to the role of psychosocial support. Higher social network support was significantly associated with greater immediate improvement in the elderly’s balance performance. This finding is consistent with prior research demonstrating that social connectedness is associated with better physical functioning, higher levels of physical activity, and improved adherence to exercise programs among older adults (Smith et al., 2017; N. Valtorta & Hanratty, 2012). Social networks may facilitate engagement by providing encouragement, accountability, and normative reinforcement of health-promoting behaviors, particularly during the early stages of an intervention. The attenuation of network effects at follow-up observed in this study is similar to findings from other behavioral interventions, where social support appears most influential during intervention initiation rather than long-term maintenance (Carron et al., 2012).

Functional support, which reflects perceived emotional, affirmational, and instrumental support, emerged as a particularly positive factor in our study. Participants with higher functional support demonstrated significantly greater immediate balance improvements, and sensitivity analyses using a more stringent cutoff revealed even larger effect sizes. These findings are consistent with the literature linking emotional support to improved psychological well-being, greater motivation, and enhanced engagement in health behaviors (Santini et al., 2020; Mendes de Leon et al., 2003). Emotional support may increase an older person’s confidence in physical capabilities and promote adaptive coping that in turn might enable responsiveness to health interventions, such as the MBB Tai Chi program. Our sensitivity analysis suggests that higher levels of perceived emotional support may confer more significant health benefit, which is consistent with the findings from a systematic review study on psychosocial resources and functional health outcomes (Uchino, 2012).

Findings related to relationship loss further underscore the importance of emotional stability in physical health intervention responsiveness. Participants reporting no recent loss of supportive relationships demonstrated greater immediate and sustained improvements in balance performance compared with those reporting relationship loss, although differences were not statistically significant. Prior research has shown that social loss and bereavement are associated with increased psychological distress, reduced physical activity, and declines in functional health among older adults (Stahl & Schulz, 2014; N. K. Valtorta et al., 2016). The pattern observed in this study suggests that disruptions to emotional support may reduce the benefits of physical health interventions, even when the intervention itself is effective.

To sum up, our findings extend prior research by demonstrating that the magnitude of balance improvement varied meaningfully according to levels of perceived social and emotional support, highlighting the importance of psychosocial context in shaping intervention effectiveness. While Tai Chi programs primarily address the elderly’s balance performance and physical health, our findings suggest that strong social networks and emotional support can potentially enhance participants’ capacity to engage with and benefit from such intervention programs. These findings may reflect the broader role of interpersonal relationships and emotional support in shaping health behaviors among older adults (Uchino, 2012). In particular, prior research has suggested that interpersonal relationships and family support may play an important role in health engagement among Latino older adults (Uchino, 2012; Smith et al., 2017; González et al., 2019). Psychosocial support may play a dual role in community-based physical activity interventions like our program. Individuals with stronger social support networks may be more likely to initiate and sustain participation in programs such as Tai Chi (Carron et al., 2012; Smith et al., 2017). At the same time, participation in group-based activities may further strengthen interpersonal relationships and emotional support among participants (Carron et al., 2012). Future interventions may benefit from intentionally incorporating strategies that promote social engagement, peer encouragement, and supportive group environments to enhance both adherence and potential health benefits (Carron et al., 2012; Uchino, 2012). While this study did not directly examine cultural mechanisms, future research could further explore how culturally meaningful social support structures influence participation in community-based health interventions (Alegria et al., 2004; González et al., 2019).

This study includes several limitations. First is the absence of a comparison or control group. Because the study employed a single-group pre–post design, the observed improvements in balance performance cannot be attributed to the Tai Chi intervention itself. Alternative explanations, such as practice effects from repeated testing of the Timed Up and Go assessment, regression to the mean, natural variation in balance ability, or increased familiarity with the testing environment and study staff, may also have contributed to the observed changes. Consequently, the findings should be interpreted as preliminary evidence of improvement among participants enrolled in the program rather than definitive causal evidence of intervention effectiveness. Future research employing randomized or controlled designs will be important for establishing causal relationships between Tai Chi participation, psychosocial support, and improvements in balance performance. Second, because the analytic sample was relatively small, interpretation of statistical significance alone may be misleading. To provide additional context, we examined standardized effect sizes for the key comparisons. The magnitude of the observed improvements ranged from moderate to large, particularly for participants with higher levels of functional psychosocial support. These findings suggest that the observed differences may have meaningful practical significance even when statistical significance was not consistently achieved. At the same time, descriptive power assessments indicated that several subgroup analyses were underpowered, which likely contributed to wide confidence intervals and the lack of statistical significance in some comparisons. Therefore, the findings should be interpreted cautiously and viewed as preliminary evidence that warrants confirmation in larger controlled studies.

Another methodological limitation relates to the categorization of psychosocial support variables using median-based cutoffs. Although dichotomizing continuous measures can reduce statistical power, this approach was used because NSSQ scores in this small sample showed substantial variability and skewness, which produced unstable estimates when modeled continuously. The median-based categorization allowed for more stable exploratory comparisons between participants with relatively higher versus lower perceived support. Nevertheless, future studies with larger samples should model psychosocial support variables continuously to better capture the full range of variation in support.

Participant inclusion in the analytic sample required completion of at least eight of the twelve intervention sessions and availability of outcome measurements. As a result, five participants who enrolled in the program were excluded from the analysis. This requirement may introduce potential selection bias if participants who attended fewer sessions differed systematically from those who completed the majority of the intervention. For example, participants who discontinued participation may have had different health statuses, mobility limitations, or levels of engagement with the program. Future studies with larger samples and more comprehensive follow-up would help evaluate potential differences between completers and non-completers. In addition, psychosocial support was assessed using the NSSQ, a self-reported measure that may be subject to recall bias or differences in how individuals perceive and report their social relationships. Although the NSSQ has demonstrated reliability and validity in prior studies, self-reported psychosocial measures may introduce measurement variability that should be considered when interpreting the findings.

## 5. Conclusions

This study adds to the existing literature by showing that psychosocial support was associated with greater improvements in balance performance among Latino participants in the Tai Chi program, particularly immediately following the intervention. However, these associations were not consistently maintained at follow-up. Our findings highlight the importance of accounting for the interactive effects of psychosocial factors when designing and implementing community-based interventions aimed at improving health and functional well-being in a growing population of older Latino adults living with chronic conditions.

## Figures and Tables

**Table 1:** Moving for Better Balance Tai Chi Intervention Program Participants’ Baseline Characteristics and Timed Up and Go Test Results (n = 23).

Characteristics	n	%	Mean ± SD	Mean TUG Seconds (95% CI) ^†^
Age	23		66.61 ± 10.74	
Younger than 60	6	30.4%	-	13.18 (7.63, 18.74)
60 years and older	17	69.6%	-	13.00 (11.34, 14.67)
Gender	23		-	
Female	16	69.6%	-	12.82 (10.89, 14.75)
Male	7	30.4%	-	13.58 (9.65, 17.50)
Social Networking Score	23		50.96 ± 23.62	
High (≥54)	12	52.2%	-	13.49 (11.05, 15.93)
Low (<54)	11	47.8%	-	12.57 (10.05, 15.09)
Functional Support Score	23		102.35 ± 49.18	
High (≥100)	12	52.2%	-	13.47 (10.76, 16.71)
Less (<100)	11	47.8%	-	12.73 (9.92, 15.53)
Total loss of relationship	23		2.57 ± 2.83	
No Loss	12	52.2%	-	12.81 (10.38, 15.23)
With Loss	11	47.8%	-	13.31 (10.74, 15.89)
Falls history	23		-	
No	16	69.6%	-	12.26 (10.38, 14.14)
Yes	7	30.4%	-	14.84 (11.29, 18.40)
Number of comorbidities ^‡^	23		2.22 ± 1.66	
None	3	13.0%	-	13.57 (6.38, 20.75)
One	6	30.4%	-	10.61 (8.48, 12.75)
Two and more	14	56.5%	-	13.98 (12.96, 14.66)
Using assisted device	23		-	
No	21	91.3%	-	12.97 (11.24, 14.71)
Yes	2	2.7%	-	13.85 (−22.36, 50.06)

^†^ TUG = Timed Up and Go Test, unit: second, CI = Confidence Interval. ^‡^ Comorbidities included stroke, cardiovascular condition, diabetes, high cholesterol, arthritis, hypertension, and cancers.

**Table 2:** Comparison of the average changes in the Timed Up and Go (TUG) immediate and 12 weeks after Moving for Better Balance Tai Chi Intervention Program by participants’ characteristics (n = 23).

Characteristics	n (%)	TUG Change After Post TC Program (95% CI) ^†^	Crude *p*-Value *	Adjusted *p*-Value **	TUG Change After 3 Months TC Program (95% CI) ^†^	Crude *p*-Value *	Adjusted *p*-Value **
Age group			0.985	0.997		0.520	0.509
Younger than 60	6 (30.4%)	0.99 (−1.17, 3.15)			0.70 (−1.46, 2.86)		
60 years and older	17 (69.6%)	0.99 (−0.38, 2.35)			1.50 (0.13, 2.87)		
Gender			0.723	0.714		0.650	0.640
Female	16 (69.7%)	1.11 (−0.27, 2.48)			1.38 (0.02, 2.75)		
Male	7 (30.3%)	1.53 (−0.47, 3.53)			1.92 (−0.08, 3.91)		
Total Networking Score			0.017 *	0.014 **		0.304	0.289
High (≥54)	12 (52.2%)	2.34 (0.59, 4.09)			2.06 (0.32, 3.79)		
Low (<54)	11 (47.8%)	−0.18 (−1.77, 1.41)			0.76 (−0.50, 2.02)		
Total Functional Score (Primary)			0.017 *	0.014 **		0.304	0.289
High (≥100)	12 (52.2%)	2.34 (0.59, 4.09)			2.06 (0.32, 3.79)		
Low (<100)	11 (47.8%)	−0.18 (−1.77, 1.41)			0.76 (−0.50, 2.02)		
Total Functional Score (Sensitive)			0.032 *	0.029 **		0.092	0.086
High (≥120)	8 (34.8%)	2.69 (0.72, 4.65)			2.64 (0.68, 4.60)		
Low (<120)	15 (65.2%)	0.27 (−1.24, 1.78)			0.74 (−0.77, 2.25)		
Total loss of relationship			0.138	0.083		0.484	0.344
No Loss	12 (52.2%)	1.93 (0.27, 3.59)			1.81 (1.18, 2.45)		
With Loss	11 (47.8%)	0.41 (−1.03, 1.85)			1.12 (0.45, 1.80)		
Falls history			0.817	---		0.236	---
No	16 (78.3%)	1.19 (−0.11, 2.49)			1.00 (−0.21, 2.21)		
Yes	7 (21.7%)	1.00 (−2.52, 4.53)			2.48 (0.05, 4.91)		
Number of comorbidities ^‡^			0.215	---		0.051	0.066
None	3 (13.0%)	2.20 (−0.31, 4.09)			3.24 (0.35, 6.14)		
One	7 (30.4%)	1.45 (−0.21, 3.12)			−0.43 (−0.91, 0.80)		
Two and more	13 (56.5%)	0.75 (−0.60, 2.10)			1.64 (1.10, 2.17)		
Using assisted device			0.371	---		0.236	---
No	21 (91.3%)	1.10 (−0.22, 2.42)			1.38 (0.21, 2.55)		
Yes	2 (2.7%)	1.51 (−22.82, 25.85)			2.14 (−22.02, 26.31)		

^†^ TUG = Timed Up and Go Test; CI = Confidence Interval. ^‡^ Comorbidities included stroke, cardiovascular condition, diabetes, high cholesterol, arthritis, hypertension, and cancers. * The significance level was set at 0.05. ** Variables were adjusted for each of the other variables in the model. The significance level was set at 0.05. Note: The categorizations for Total Networking Score and Total Functional Support Score (Primary) were identical due to the median-based cutoff approach used in this sample.

## Data Availability

The data used in this study are available from the corresponding author upon reasonable request.

## Abbreviations

The following abbreviations are used in this manuscript:

MBB	Moving for Better Balance
TUG	Timed Up and Go Test
NSSQ	Norbeck Social Support Questionnaire
CI	Confidence Interval
